# *N*′-[(*E*)-1-(3,5-Dichloro-2-hy­droxy­phen­yl)ethyl­idene]-4-meth­oxy­benzo­hydrazide monohydrate

**DOI:** 10.1107/S1600536810038328

**Published:** 2010-09-30

**Authors:** Chun-Hong He, Jian-Ping Zhang, Jian-Guo Chang

**Affiliations:** a Department of Materials Science and chemical Engineering, Taishan University, 271021 Taian, Shandong, People’s Republic of China

## Abstract

The title compound, C_16_H_14_Cl_2_N_2_O_3_·H_2_O, displays a *trans* conformation with respect to the C=N double bond. The dihedral angle between the two benzene rings is 4.98 (12)°. Intra­molecular O—H⋯N and O—H⋯O hydrogen bonds occur. The crystal structure is stabilized by inter­molecular O—H⋯O and N—H⋯O hydrogen bonds. In addition, there are π–π inter­actions between the chemically distinct benzene rings of inversion-related mol­ecules [centroid–centroid separation = 3.715 (1) Å].

## Related literature

For further details of the chemistry of the title compound, see: Carcelli *et al.* (1995 ▶); Salem (1998 ▶). For a related stucture, see: Chang *et al.* (2007 ▶).
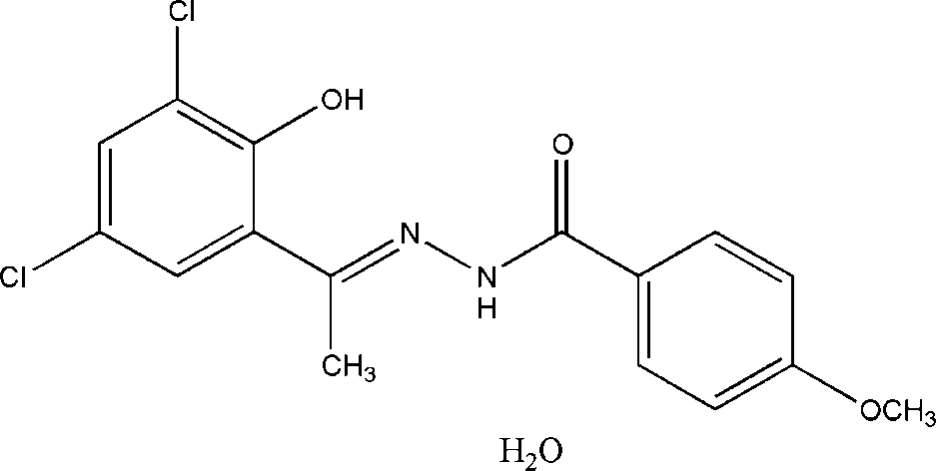


## Experimental

### 

#### Crystal data

C_16_H_14_Cl_2_N_2_O_3_·H_2_O
*M* *_r_* = 371.21
Triclinic, 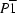

*a* = 7.033 (5) Å
*b* = 7.516 (7) Å
*c* = 16.647 (10) Å
α = 85.105 (10)°
β = 81.386 (12)°
γ = 79.414 (10)°
*V* = 853.7 (11) Å^3^
*Z* = 2
Mo *K*α radiation
μ = 0.40 mm^−1^
*T* = 298 K
0.30 × 0.23 × 0.16 mm

#### Data collection

Bruker APEXII CCD area-detector diffractometer
Absorption correction: multi-scan (*SADABS*; Sheldrick, 2003 ▶) *T*_min_ = 0.906, *T*_max_ = 0.946
4423 measured reflections
2936 independent reflections
1997 reflections with *I* > 2σ(*I*)
*R*_int_ = 0.025

#### Refinement

*R*[*F* ^2^ > 2σ(*F* ^2^)] = 0.066
*wR*(*F* ^2^) = 0.170
*S* = 1.00
2936 reflections
220 parameters
H-atom parameters constrained
Δρ_max_ = 0.56 e Å^−3^
Δρ_min_ = −0.32 e Å^−3^

### 

Data collection: *APEX2* (Bruker, 2005 ▶); cell refinement: *SAINT* (Bruker, 2005 ▶); data reduction: *SAINT*; program(s) used to solve structure: *SHELXS97* (Sheldrick, 2008 ▶); program(s) used to refine structure: *SHELXL97* (Sheldrick, 2008 ▶); molecular graphics: *SHELXTL* (Sheldrick, 2008 ▶); software used to prepare material for publication: *SHELXTL*.

## Supplementary Material

Crystal structure: contains datablocks global, I. DOI: 10.1107/S1600536810038328/pk2270sup1.cif

Structure factors: contains datablocks I. DOI: 10.1107/S1600536810038328/pk2270Isup2.hkl

Additional supplementary materials:  crystallographic information; 3D view; checkCIF report

## Figures and Tables

**Table 1:** Hydrogen-bond geometry (Å, °)

*D*—H⋯*A*	*D*—H	H⋯*A*	*D*⋯*A*	*D*—H⋯*A*
N2—H2⋯O4	0.86	2.15	2.926 (5)	150
O1—H1⋯O2	0.82	2.58	3.287 (5)	146
O1—H1⋯N1	0.82	1.77	2.484 (5)	145
O4—H16⋯O1^i^	0.85	2.09	2.887 (5)	156
O4—H15⋯O2^ii^	0.85	1.88	2.726 (5)	176

Symmetry codes: (i) 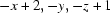; (ii) 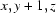.

**Fig. 1.**
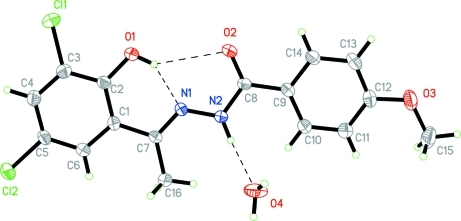
The molecular structure of compound, showing the atom-numbering scheme. Displacement ellipsoids are drawn at the 30° probability level. Dashed lines show intramolecular O—H···N, O—H···O and intermolecular N—H···O hydrogen bonds.

**Fig. 2.**
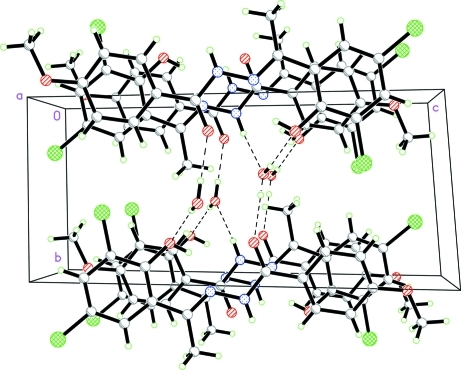
Packing diagram of compound, Showing intermolecular O—H···O and N—H···O hydrogen bonds (dashed lines).

## supplementary crystallographic information

### Comment

The chemistry of aroylhydrazones continues to attract much attention due to their coordination ability to metal ions and their biological activity (Carcelli *et al.*, 1995; Salem, 1998; Chang *et al.*, 2007).  As an extension of work on the structural characterization of aroylhydrazone derivatives, the title compound, was synthesized and its crystal structure is reported here.

The title molecule displays a *trans* conformation with respect to the C7=N1 double bond (Fig. 1). The dihedral angle between the two benzene rings is 4.98 (12) °. The crystal structure is stabilized by intramolecular O—H···N, O—H···O and intermolecular O—H···O, N—H···O hydrogen bonds. (Table. 1, Figs. 1 and 2).  There are π - π interactions between the chemically distinct benzene rings on inversion related molecules [*Cg*···*Cg* = 3.715 (1) Å; *Cg* represents a ring centroid].

### Experimental

4-methoxybenzohydrazide (0.01 mol,1.66 g) was dissolved in anhydrous ethanol (50 ml), and 1-(3,5-dichloro-2-hydroxyphenyl)ethanone (0.01 mol, 2.05 g) was added. The reaction mixture was refluxed for 5 h with stirring, then the resulting precipitate was collected by filtration, washed several times with ethanol and dried *in vacuo* (yield 78%). The compound (1.0 mmol,0.35 g) was dissolved in dimethylformamide (30 ml) and kept at room temperature for 20d to obtain yellow single crystals suitable for X-ray diffraction.

### Refinement

All H atoms were positioned geometrically and treated as riding on their parent atoms, with C—H (methyl) = 0.96 Å, C—H (aromatic) = 0.93 Å, O—H = 0.82 Å, N—H = 0.86 Å and with *U*_iso_(H) = 1.5*U*_eq_(C_methyl_, O and 1.2*U*_eq_(C_aromatic_, C_methylene_, N).

### Crystal data

**Table:**

C_16_H_14_Cl_2_N_2_O_3_·H_2_O	*Z* = 2
*M**_r_* = 371.21	*F*(000) = 384
Triclinic, *P*1	*D*_x_ = 1.444 Mg m^−^^3^
Hall symbol: -P 1	Mo *K*α radiation, λ = 0.71073 Å
*a* = 7.033 (5) Å	Cell parameters from 1429 reflections
*b* = 7.516 (7) Å	θ = 3.0–25.5°
*c* = 16.647 (10) Å	µ = 0.40 mm^−^^1^
α = 85.105 (10)°	*T* = 298 K
β = 81.386 (12)°	Plate, yellow
γ = 79.414 (10)°	0.30 × 0.23 × 0.16 mm
*V* = 853.7 (11) Å^3^	

### Data collection

**Table:**

Bruker APEXII CCD area-detector diffractometer	2936 independent reflections
Radiation source: fine-focus sealed tube	1997 reflections with *I* > 2σ(*I*)
graphite	*R*_int_ = 0.025
φ and ω scans	θ_max_ = 25.1°, θ_min_ = 2.5°
Absorption correction: multi-scan (*SADABS*; Sheldrick, 2003)	*h* = −8→8
*T*_min_ = 0.906, *T*_max_ = 0.946	*k* = −6→8
4423 measured reflections	*l* = −19→19

### Refinement

**Table:**

Refinement on *F*^2^	Primary atom site location: structure-invariant direct methods
Least-squares matrix: full	Secondary atom site location: difference Fourier map
*R*[*F*^2^ > 2σ(*F*^2^)] = 0.066	Hydrogen site location: inferred from neighbouring sites
*wR*(*F*^2^) = 0.170	H-atom parameters constrained
*S* = 1.00	*w* = 1/[σ^2^(*F*_o_^2^) + (0.0727*P*)^2^ + 0.8688*P*] where *P* = (*F*_o_^2^ + 2*F*_c_^2^)/3
2936 reflections	(Δ/σ)_max_ < 0.001
220 parameters	Δρ_max_ = 0.56 e Å^−^^3^
0 restraints	Δρ_min_ = −0.32 e Å^−^^3^

### Special details

**Table:**

Geometry. All e.s.d.'s (except the e.s.d. in the dihedral angle between two l.s. planes) are estimated using the full covariance matrix. The cell e.s.d.'s are taken into account individually in the estimation of e.s.d.'s in distances, angles and torsion angles; correlations between e.s.d.'s in cell parameters are only used when they are defined by crystal symmetry. An approximate (isotropic) treatment of cell e.s.d.'s is used for estimating e.s.d.'s involving l.s. planes.
Refinement. Refinement of *F*^2^ against ALL reflections. The weighted *R*-factor *wR* and goodness of fit *S* are based on *F*^2^, conventional *R*-factors *R* are based on *F*, with *F* set to zero for negative *F*^2^. The threshold expression of *F*^2^ > σ(*F*^2^) is used only for calculating *R*-factors(gt) *etc*. and is not relevant to the choice of reflections for refinement. *R*-factors based on *F*^2^ are statistically about twice as large as those based on *F*, and *R*- factors based on ALL data will be even larger.

### Fractional atomic coordinates and isotropic or equivalent isotropic displacement parameters (Å^2^)

**Table:**

	*x*	*y*	*z*	*U*_iso_*/*U*_eq_	
Cl1	1.0655 (2)	−0.37144 (18)	0.19059 (9)	0.0675 (5)	
Cl2	0.8926 (3)	0.3075 (2)	0.05875 (8)	0.0774 (6)	
O1	0.9149 (5)	−0.2090 (4)	0.34325 (19)	0.0462 (8)	
H1	0.8675	−0.1599	0.3856	0.069*	
O2	0.7613 (6)	−0.2102 (4)	0.5397 (2)	0.0611 (10)	
O3	0.5111 (6)	0.0452 (6)	0.8985 (2)	0.0714 (12)	
O4	0.7853 (6)	0.4252 (4)	0.5716 (3)	0.0710 (12)	
N1	0.7805 (5)	0.0497 (5)	0.4303 (2)	0.0367 (9)	
N2	0.7259 (5)	0.0879 (5)	0.5112 (2)	0.0392 (9)	
H2	0.6983	0.1973	0.5264	0.047*	
C1	0.8427 (6)	0.1022 (6)	0.2902 (3)	0.0360 (9)	
C2	0.9122 (6)	−0.0844 (6)	0.2809 (3)	0.0374 (10)	
C3	0.9789 (7)	−0.1428 (6)	0.2023 (3)	0.0442 (11)	
C4	0.9757 (7)	−0.0251 (7)	0.1341 (3)	0.0488 (11)	
H4	1.0221	−0.0668	0.0826	0.059*	
C5	0.9020 (7)	0.1566 (7)	0.1441 (3)	0.0448 (11)	
C6	0.8384 (7)	0.2200 (6)	0.2203 (3)	0.0419 (10)	
H6	0.7918	0.3432	0.2255	0.050*	
C7	0.7781 (6)	0.1720 (6)	0.3720 (3)	0.0357 (9)	
C8	0.7186 (6)	−0.0562 (6)	0.5645 (3)	0.0387 (10)	
C9	0.6577 (6)	−0.0211 (6)	0.6511 (3)	0.0387 (10)	
C10	0.5662 (7)	0.1480 (7)	0.6775 (3)	0.0452 (11)	
H10	0.5393	0.2442	0.6396	0.054*	
C11	0.5141 (7)	0.1750 (7)	0.7606 (3)	0.0508 (12)	
H11	0.4542	0.2887	0.7780	0.061*	
C12	0.5526 (7)	0.0310 (7)	0.8165 (3)	0.0483 (11)	
C13	0.6383 (7)	−0.1378 (7)	0.7914 (3)	0.0517 (12)	
H13	0.6614	−0.2346	0.8294	0.062*	
C14	0.6904 (7)	−0.1634 (7)	0.7088 (3)	0.0457 (11)	
H14	0.7484	−0.2780	0.6919	0.055*	
C15	0.4310 (10)	0.2206 (10)	0.9292 (4)	0.0819 (19)	
H15A	0.5202	0.3029	0.9109	0.123*	
H15B	0.4107	0.2103	0.9876	0.123*	
H15C	0.3086	0.2657	0.9095	0.123*	
C16	0.7148 (6)	0.3729 (4)	0.3805 (3)	0.0605 (15)	
H16A	0.6850	0.3969	0.4371	0.091*	
H16B	0.6008	0.4148	0.3542	0.091*	
H16C	0.8183	0.4351	0.3555	0.091*	
H15	0.7839	0.5382	0.5611	0.14 (3)*	
H16	0.8905	0.3884	0.5919	0.09 (2)*	

### Atomic displacement parameters (Å^2^)

**Table:**

	*U*^11^	*U*^22^	*U*^33^	*U*^12^	*U*^13^	*U*^23^
Cl1	0.0958 (12)	0.0380 (7)	0.0647 (9)	−0.0048 (7)	−0.0002 (8)	−0.0142 (6)
Cl2	0.1369 (15)	0.0568 (9)	0.0331 (7)	−0.0125 (9)	−0.0067 (8)	0.0091 (6)
O1	0.067 (2)	0.0312 (17)	0.0383 (18)	−0.0054 (15)	−0.0049 (16)	0.0012 (14)
O2	0.104 (3)	0.0296 (18)	0.046 (2)	−0.0090 (18)	−0.0052 (19)	0.0065 (15)
O3	0.089 (3)	0.084 (3)	0.034 (2)	−0.001 (2)	−0.0038 (19)	0.0013 (19)
O4	0.090 (3)	0.037 (2)	0.096 (3)	−0.0169 (19)	−0.044 (3)	0.007 (2)
N1	0.050 (2)	0.031 (2)	0.0279 (19)	−0.0080 (16)	−0.0040 (16)	0.0009 (15)
N2	0.053 (2)	0.0283 (19)	0.035 (2)	−0.0060 (16)	−0.0037 (17)	−0.0006 (16)
C1	0.038 (2)	0.039 (2)	0.032 (2)	−0.0095 (18)	−0.0057 (17)	−0.0004 (17)
C2	0.041 (2)	0.033 (2)	0.038 (2)	−0.0108 (18)	−0.0052 (18)	0.0006 (18)
C3	0.048 (2)	0.041 (2)	0.045 (2)	−0.0085 (19)	−0.005 (2)	−0.007 (2)
C4	0.057 (3)	0.049 (3)	0.040 (2)	−0.013 (2)	−0.003 (2)	−0.006 (2)
C5	0.058 (3)	0.043 (2)	0.034 (2)	−0.013 (2)	−0.007 (2)	0.0028 (19)
C6	0.048 (2)	0.038 (2)	0.038 (2)	−0.0076 (19)	−0.0049 (19)	0.0003 (19)
C7	0.043 (2)	0.031 (2)	0.033 (2)	−0.0059 (17)	−0.0058 (18)	0.0025 (17)
C8	0.047 (2)	0.029 (2)	0.039 (2)	−0.0067 (18)	−0.0073 (19)	0.0035 (18)
C9	0.040 (2)	0.040 (2)	0.037 (2)	−0.0101 (18)	−0.0062 (18)	0.0037 (18)
C10	0.051 (3)	0.043 (2)	0.039 (2)	−0.005 (2)	−0.005 (2)	0.002 (2)
C11	0.057 (3)	0.047 (3)	0.046 (3)	−0.006 (2)	−0.002 (2)	−0.002 (2)
C12	0.048 (3)	0.059 (3)	0.037 (2)	−0.008 (2)	−0.005 (2)	0.001 (2)
C13	0.051 (3)	0.058 (3)	0.043 (2)	−0.006 (2)	−0.008 (2)	0.011 (2)
C14	0.050 (3)	0.044 (2)	0.041 (2)	−0.006 (2)	−0.005 (2)	0.006 (2)
C15	0.100 (5)	0.092 (5)	0.048 (3)	−0.004 (4)	0.003 (3)	−0.018 (3)
C16	0.098 (4)	0.035 (3)	0.041 (3)	−0.001 (3)	−0.001 (3)	−0.001 (2)

### Geometric parameters (Å, °)

**Table:**

Cl1—C3	1.731 (5)	C5—C6	1.376 (7)
Cl2—C5	1.742 (5)	C6—H6	0.9300
O1—C2	1.338 (5)	C7—C16	1.506 (5)
O1—H1	0.8200	C8—C9	1.474 (6)
O2—C8	1.231 (5)	C9—C14	1.385 (6)
O3—C12	1.362 (6)	C9—C10	1.391 (6)
O3—C15	1.440 (7)	C10—C11	1.399 (7)
O4—H15	0.8511	C10—H10	0.9300
O4—H16	0.8496	C11—C12	1.381 (7)
N1—C7	1.279 (5)	C11—H11	0.9300
N1—N2	1.383 (5)	C12—C13	1.372 (7)
N2—C8	1.344 (5)	C13—C14	1.389 (7)
N2—H2	0.8600	C13—H13	0.9300
C1—C6	1.401 (6)	C14—H14	0.9300
C1—C2	1.412 (6)	C15—H15A	0.9600
C1—C7	1.477 (6)	C15—H15B	0.9600
C2—C3	1.401 (6)	C15—H15C	0.9600
C3—C4	1.378 (7)	C16—H16A	0.9600
C4—C5	1.383 (7)	C16—H16B	0.9600
C4—H4	0.9300	C16—H16C	0.9600
			
C2—O1—H1	109.5	C14—C9—C10	118.5 (4)
C12—O3—C15	118.7 (5)	C14—C9—C8	118.6 (4)
H15—O4—H16	104.1	C10—C9—C8	122.9 (4)
C7—N1—N2	123.2 (4)	C9—C10—C11	120.7 (5)
C8—N2—N1	115.9 (4)	C9—C10—H10	119.7
C8—N2—H2	122.0	C11—C10—H10	119.7
N1—N2—H2	122.0	C12—C11—C10	119.3 (5)
C6—C1—C2	118.5 (4)	C12—C11—H11	120.4
C6—C1—C7	120.8 (4)	C10—C11—H11	120.4
C2—C1—C7	120.7 (4)	O3—C12—C13	115.8 (5)
O1—C2—C3	118.2 (4)	O3—C12—C11	123.4 (5)
O1—C2—C1	123.3 (4)	C13—C12—C11	120.8 (5)
C3—C2—C1	118.5 (4)	C12—C13—C14	119.6 (5)
C4—C3—C2	122.3 (4)	C12—C13—H13	120.2
C4—C3—Cl1	119.1 (4)	C14—C13—H13	120.2
C2—C3—Cl1	118.6 (4)	C9—C14—C13	121.2 (5)
C3—C4—C5	118.5 (4)	C9—C14—H14	119.4
C3—C4—H4	120.8	C13—C14—H14	119.4
C5—C4—H4	120.8	O3—C15—H15A	109.5
C6—C5—C4	121.1 (4)	O3—C15—H15B	109.5
C6—C5—Cl2	119.6 (4)	H15A—C15—H15B	109.5
C4—C5—Cl2	119.3 (4)	O3—C15—H15C	109.5
C5—C6—C1	121.1 (4)	H15A—C15—H15C	109.5
C5—C6—H6	119.5	H15B—C15—H15C	109.5
C1—C6—H6	119.5	C7—C16—H16A	109.5
N1—C7—C1	114.5 (4)	C7—C16—H16B	109.5
N1—C7—C16	125.9 (4)	H16A—C16—H16B	109.5
C1—C7—C16	119.6 (4)	C7—C16—H16C	109.5
O2—C8—N2	119.6 (4)	H16A—C16—H16C	109.5
O2—C8—C9	122.8 (4)	H16B—C16—H16C	109.5
N2—C8—C9	117.6 (4)		
			
C7—N1—N2—C8	173.8 (4)	C6—C1—C7—C16	−2.6 (6)
C6—C1—C2—O1	−177.2 (4)	C2—C1—C7—C16	176.7 (4)
C7—C1—C2—O1	3.5 (6)	N1—N2—C8—O2	0.7 (6)
C6—C1—C2—C3	1.7 (6)	N1—N2—C8—C9	−179.0 (4)
C7—C1—C2—C3	−177.5 (4)	O2—C8—C9—C14	14.7 (7)
O1—C2—C3—C4	177.9 (4)	N2—C8—C9—C14	−165.6 (4)
C1—C2—C3—C4	−1.1 (7)	O2—C8—C9—C10	−164.4 (5)
O1—C2—C3—Cl1	−1.4 (6)	N2—C8—C9—C10	15.3 (6)
C1—C2—C3—Cl1	179.7 (3)	C14—C9—C10—C11	2.0 (7)
C2—C3—C4—C5	−0.7 (7)	C8—C9—C10—C11	−178.9 (4)
Cl1—C3—C4—C5	178.5 (4)	C9—C10—C11—C12	−0.7 (7)
C3—C4—C5—C6	1.9 (7)	C15—O3—C12—C13	176.6 (5)
C3—C4—C5—Cl2	−178.9 (4)	C15—O3—C12—C11	−2.9 (8)
C4—C5—C6—C1	−1.3 (7)	C10—C11—C12—O3	178.5 (5)
Cl2—C5—C6—C1	179.6 (4)	C10—C11—C12—C13	−1.0 (8)
C2—C1—C6—C5	−0.6 (7)	O3—C12—C13—C14	−178.2 (4)
C7—C1—C6—C5	178.7 (4)	C11—C12—C13—C14	1.3 (8)
N2—N1—C7—C1	179.8 (4)	C10—C9—C14—C13	−1.7 (7)
N2—N1—C7—C16	−0.8 (7)	C8—C9—C14—C13	179.2 (4)
C6—C1—C7—N1	176.9 (4)	C12—C13—C14—C9	0.1 (7)
C2—C1—C7—N1	−3.9 (6)		

### Hydrogen-bond geometry (Å, °)

**Table:**

*D*—H···*A*	*D*—H	H···*A*	*D*···*A*	*D*—H···*A*
N2—H2···O4	0.86	2.15	2.926 (5)	150
O1—H1···O2	0.82	2.58	3.287 (5)	146
O1—H1···N1	0.82	1.77	2.484 (5)	145
O4—H16···O1^i^	0.85	2.09	2.887 (5)	156
O4—H15···O2^ii^	0.85	1.88	2.726 (5)	176

Symmetry codes: (i) −*x*+2, −*y*, −*z*+1; (ii) *x*, *y*+1, *z*.
